# US10 Protein Is Crucial but not Indispensable for Duck Enteritis Virus Infection in Vitro

**DOI:** 10.1038/s41598-018-34503-7

**Published:** 2018-11-07

**Authors:** Yunchao Ma, Qiurui Zeng, Mingshu Wang, Anchun Cheng, Renyong Jia, Qiao Yang, Ying Wu, Xin-Xin Zhao, Mafeng Liu, Dekang Zhu, Shun Chen, Shaqiu Zhang, Yunya Liu, Yanling Yu, Ling Zhang, Xiaoyue Chen

**Affiliations:** 1 Institute of Preventive Veterinary Medicine, Sichuan Agricultural University, Wenjiang, Chengdu City, Sichuan 611130 P.R. China; 2 Key Laboratory of Animal Disease and Human Health of Sichuan Province, Sichuan Agricultural University, Wenjiang, Chengdu City, Sichuan 611130 P.R. China; 3 Avian Disease Research Center, College of Veterinary Medicine, Sichuan Agricultural University, Wenjiang, Chengdu City, Sichuan 611130 P.R. China; 4 School of Medicine, Shanghai Jiao Tong University, Shanghai, 200025 P.R. China

## Abstract

To investigate the function of the duck enteritis virus (DEV) tegument protein US10, we generated US10 deletion and revertant mutants (ΔUS10 and US10FRT) via two-step RED recombination based on an infectious BAC clone of DEV CHv-BAC-G (BAC-G). In multistep growth kinetic analyses, ΔUS10 showed an approximately 100-fold reduction in viral titer, while the genome copies decreased only 4-fold compared to those of BAC-G. In one-step growth kinetic analyses, there were no significant differences in genome copies among BAC-G, ΔUS10 and US10FRT, but ΔUS10 still showed a 5- to 20-fold reduction in viral titer, and the replication defect of ΔUS10 was partially reversed by infection of US10-expressing cells. The transcription levels of Mx, OASL, IL-4, IL-6 and IL-10 in ΔUS10-infected duck embryo fibroblasts (DEFs) were significantly upregulated, while TLR3 was downregulated compared with those in BAC-G-infected DEFs. Taken together, these data indicated that US10 is vital for DEV replication and is associated with transcription of some immunity genes.

## Introduction

Herpesviruses are classified into three subfamilies, designated alpha-, beta- and gammaherpesviruses, all of which can establish lifelong latent infections^1^. Alphaherpesviruses are primarily distinguished by higher reproductive efficiency than other subfamily members. In animal virology, alphaherpesviruses are important pathogens responsible for many acute or chronic diseases. As a member of the alphaherpesviruses, duck enteritis virus (DEV), also known as duck plague virus (DPV), is the causative agent of duck enteritis, an acute, contagious disease of waterfowl^2,3^ that results in significant losses in domestic and wild waterfowl due to high mortality^4–7^.

Herpesvirus virions consist of four morphologically distinct structures, the linear double-stranded DNA, capsid, tegument and envelope^2,8–11^. Herpes simplex virus type 1 (HSV-1) replicates its genome in the nucleus^1^. The nucleocapsid is transported over a long distance from the cytoplasm to the nuclear pore, which is facilitated by the tegument, a complex protein-rich layer between the envelope and capsid^1,9^. In addition, tegument proteins mediate other diverse functions during the viral life cycle, such as regulation of the host cell immune system^12^, tegumentation and secondary envelopment^1,13^. Members of the tegument layer are host-cell molecules and viral-encoded proteins^13–15^, indicating that the interplay between tegument proteins and host cells is very close and complex. Compared to research on other herpesviruses, such as HSV-1, progress in DEV molecular biology research is slow. To date, only basic characteristics of some DEV genes have been reported^16–48^. To our knowledge, the role of the DEV tegument proteins in the viral life cycle has not been characterized. The focus of the experiments described here is DEV US10, a poorly understood tegument protein.

Homologs of US10 are found in many other alphaherpesviruses, and the US10 gene of HSV-1 encodes a polypeptide of 313 amino acids, which is located mainly in the nuclear matrix as a capsid/tegument-associated phosphoprotein^15^. However, the functions of US10 proteins in viral replication and infection are not well understood. Previously, we found that the DEV genome sequences of virulent and attenuated strains showed a remarkable diversity in the US10 region, and virulent strains (CHv, 2085 and CSC) have a region that is approximately 150 bp longer than those of attenuated strains (C-KCE, VAC, Clone-03, CV and K)^49–54^, suggesting that US10 might be associated with virulence. However, the role of US10 in DEV replication is still unclear. Recombinant genetic engineering techniques have led to advances in molecular biology studies of DEV^55–58^, and the bacterial artificial chromosome (BAC), the genetic technique we used in this study, is considered a powerful tool for generating recombinant mutants to study the biology and pathogenesis of herpesviruses. Zinc finger proteins, characterized by zinc finger structural motifs, are generally known as DNA- and RNA-binding factors^59,60^. The 13 amino acid sequence (C-X3-C-X3-H-X3-C) encoded by DEV US10 matches the CCHC-type zinc finger domain^22^, but the function of zinc finger proteins in this virus remains unclear.

To gain insight into the function of DEV US10, we generated US10 deletion and revertant mutants based on an infectious BAC clone of the DEV Chinese virulent (CHv) strain^58^. Then, the replication kinetics of recombinant viruses were determined to investigate the function of US10 during infection in cell culture. Furthermore, to determine whether DEV US10 plays a role in immune regulation, we measured the transcription levels of some immune-related genes in virus-infected DEFs by relative real-time quantitative PCR analyses.

## Results

### Construction and identification of recombinant pDEV-BACs

**Figure 1:**
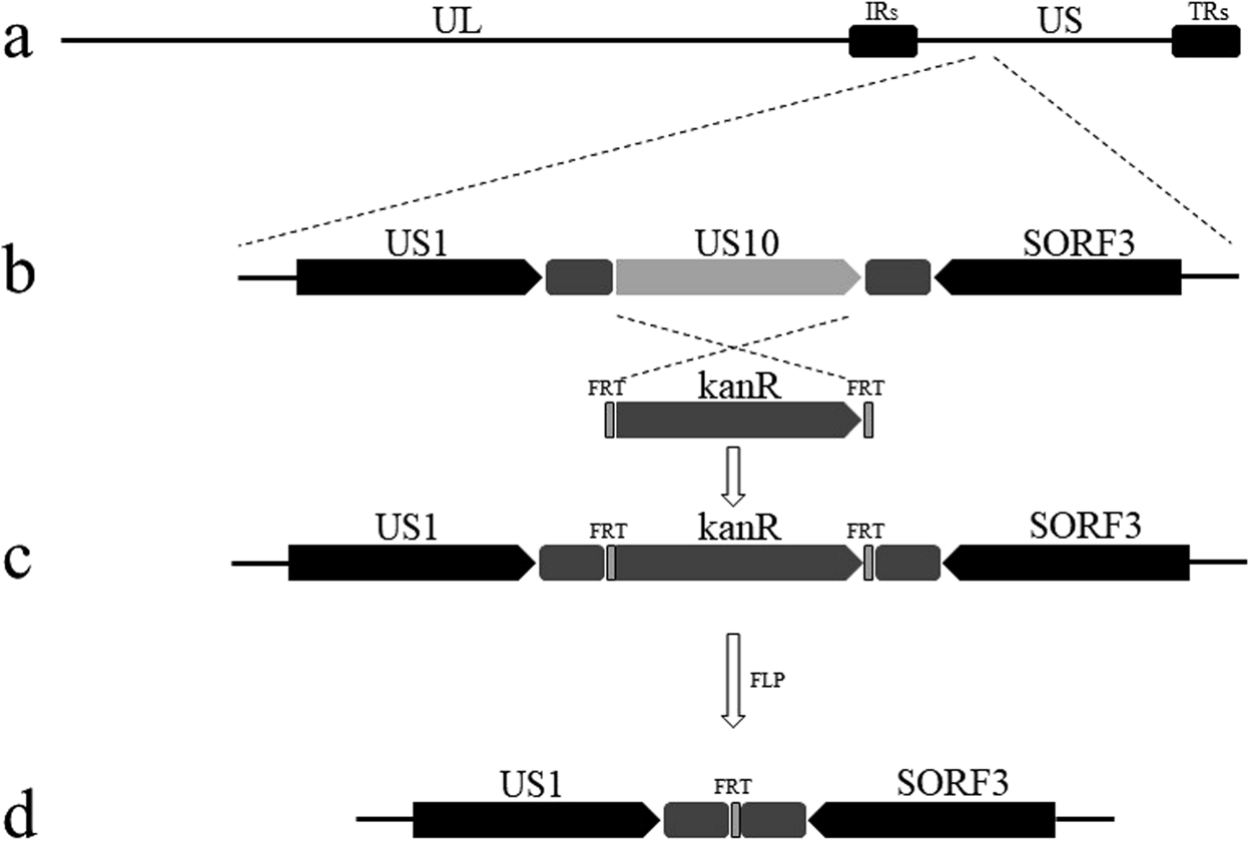
Schematic diagrams of US10 deletion. (**a**) The DEV genome consists of unique long (UL), unique short (US), internal repeat (IR) and terminal repeat (TR) regions. (**b**) Partial US region. (**c**) US10 ORF is replaced by kanR. (**d**) KanR deletion by the Flp-FRT recombination system.

**Figure 2:**
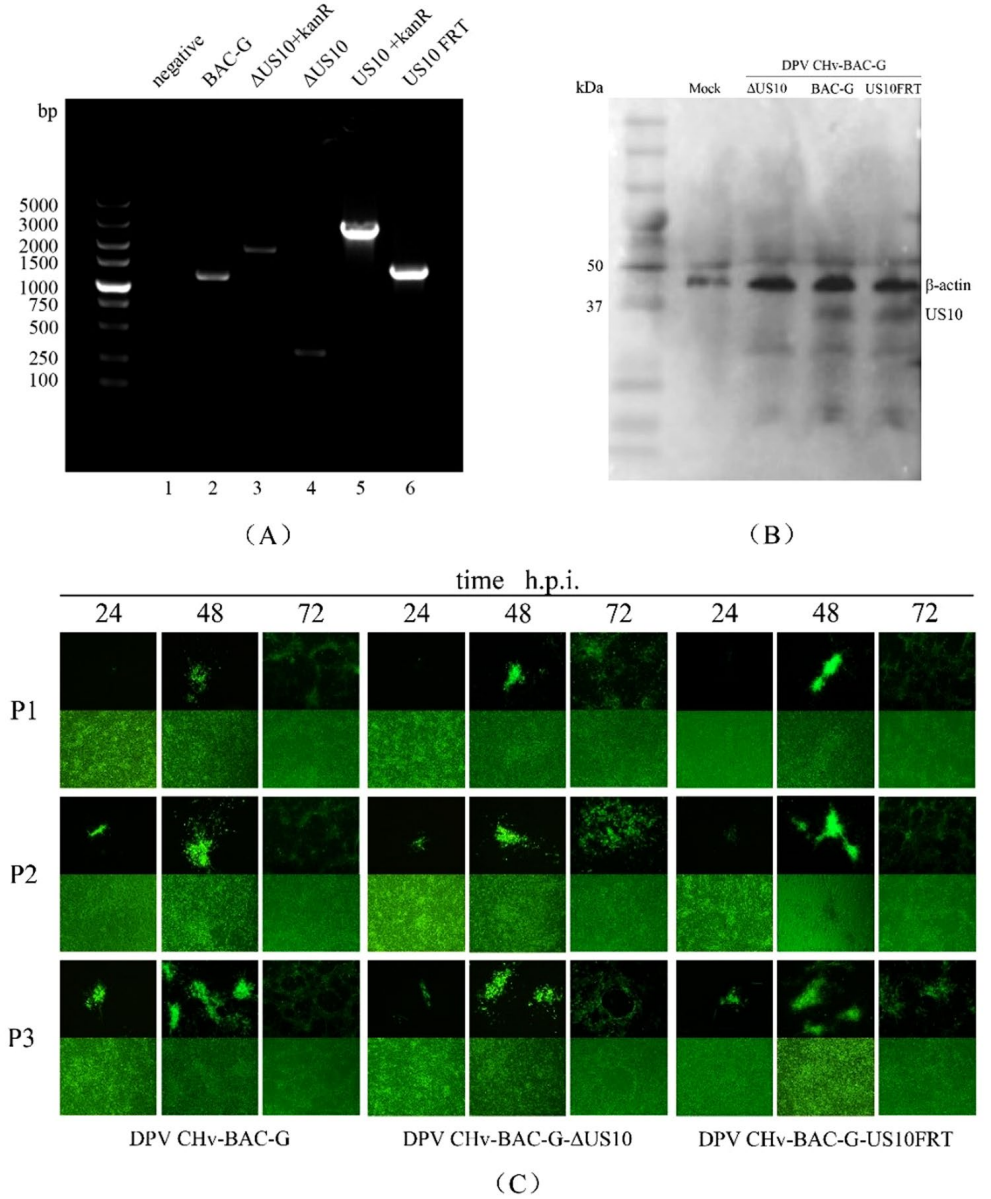
Construction and identification of parental and recombinant viruses. (**A**) PCR analysis of recombinant BACs. The BAC DNAs of BAC-G, ΔUS10 + kanR, ΔUS10, US10 + kanR and US10FRT were extracted and amplified by PCR using the indicated primers. (**B**) Western blot analyses of recombinant viruses. Anti-US10 monoclonal antibody(MAb) and anti-β-actin Mab were used to detect US10 and β-actin in total proteins extracted from mock-, ΔUS10-, BAC-G or US10FRT-infected DEFs. (**C**) Passage of rescued viruses in DEFs. Enrichment of rescued viruses were obtained by the three times passage after transfection.

The US10 deletion and revertant mutants were constructed via two-step RED recombination based on an infectious DEV BAC clone (pDEV-BAC), as described in the Materials and Methods. The entire US10 ORF was knocked out from pDEV-BAC, within which an FRT site was left (Fig. 1). To exclude the possibility that the FRT site might have an unexpected effect during viral replication, we also constructed the US10-revertant mutant BAC with an FRT site downstream of the US10 ORF. Recombinant BACs were confirmed by PCR analysis using specific primers targeting US10 flanking non-encoding sequences (Fig. 2A). *Escherichia coli* clones containing corresponding BACs were used as templates. As expected, five DNA bands of approximately 1200, 1900, 320, 2700 and 1300 bp in length were amplified separately, and the corresponding products were US10, kanR, US10 flanking sequence, US10-kanR and US10FRT (Fig. 2A, lanes 2–6). No band was detected in the negative control group (Fig. 2A, lane 1 and Supplementary Fig.). These results showed that the ΔUS10 and US10FRT mutants were constructed successfully.

### Virus rescue and identification

BAC DNAs were extracted using the Qiagen Plasmid Midi Kit and transfected into DEFs. DEFs were cultured for 5–7 days, and high levels of green fluorescence with matching cytopathic effect (CPE) were observed, indicating that BAC-G, ΔUS10 and US10FRT were rescued and generated successfully. For advanced identification of US10 expression, total proteins from mock- or virus-infected DEFs were harvested separately for western blotting. US10 expression was detected in both parental and revertant virus-infected DEFs but not in deletion mutant-infected DEFs (Fig. 2B and Supplementary Fig.). Meanwhile, rescued viruses were passaged in DEFs at least 3 times before follow-up experiments (Fig. 2C).

### Viral multistep growth kinetic analyses

**Figure 3:**
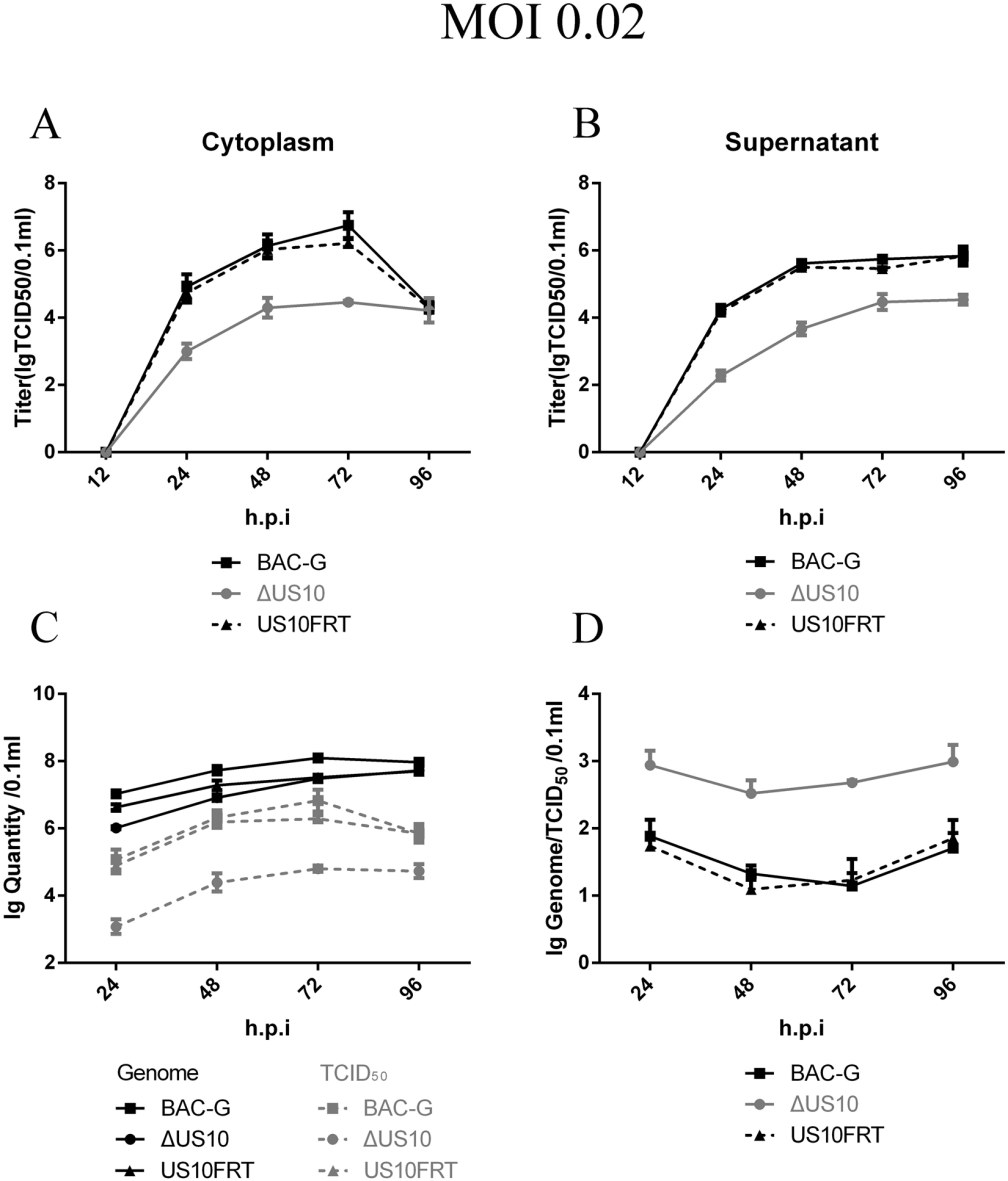
Viral titer and genome copies in multistep growth kinetics. Confluent DEF cells monolayers were infected with each virus shown at an MOI of 0.02. Viral titer and viral copies of infected supernatant, cells and mixture of cells cultures were determined at the indicated time points by measuring TCID50 on DEF cells. All titrations were carried out in three independent experiment. The titers and copies obtained were averaged, and the standard error of the mean was calculated each time point. (**A**) Viral titer in cytoplasm samples. (**B**) Viral titer in supernatant samples. (**C**) Total viral titer and genome copies. (**D**) Genome/TCID_50_ ratios.

To investigate the role of US10 in the viral replication cycle, we performed multistep replication analyses of BAC-G, ΔUS10 and US10FRT as described in the Materials and Methods. DEFs were infected with the corresponding viruses at an MOI of 0.02. At the early stage of infection, almost all incubated viruses entered the cells and initiated the replication cycle, and no infectious virions were detected at 12 h post-infection (h.p.i). After 24 h.p.i, the viral titer in the supernatant continued to increase and maintained a high level at 96 h.p.i, indicating that the deletion of US10 causes no defect in viral release (Fig. 3B). The viral titer of the cytoplasm stopped increasing and declined at 96 h.p.i because the cells were dying (Fig. 3A). The viral titer of the US10 deletion mutant showed a significant decrease compared to that of the parental and revertant strains during a 72 h period, and an approximately 100-fold growth defect in the viral titer of the US10 deletion mutant was detected at 48 h.p.i (Fig. 3C). These results suggested that US10 plays an important role in viral replication.

To investigate which stage of viral replication was blocked by the deletion of US10, we used qPCR to determine the number of viral genomes within cytoplasmic and extracellular samples (Fig. 3C). Viral DNA copies among BAC-G, US10FRT and ΔUS10 showed lower significant differences compared to those of viral titer, and copies reached the same level at 96 h.p.i. Furthermore, the genome/TCID_50_ ratios of ΔUS10 were substantially higher than those of BAC-G and US10FRT, indicating that US10 might be associated with viral maturation. These observations prompted us to further investigate viral DNA and infectious virion replication, and then, one-step replication analyses were carried out.

### Viral one-step growth kinetic analyses

**Figure 4:**
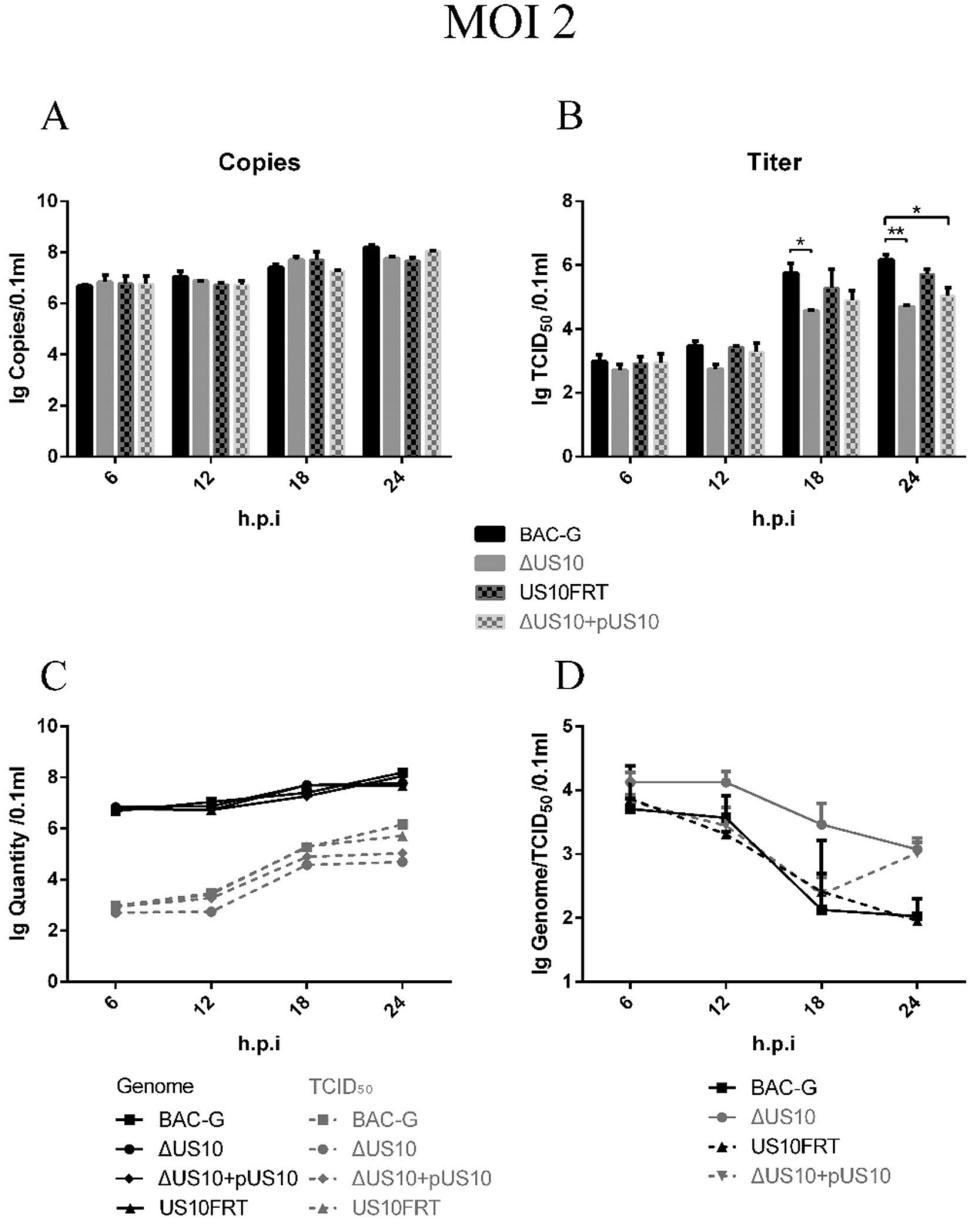
Viral titer and genome copies in one-step growth kinetics. Confluent DEF cells monolayers were infected with each virus shown at an MOI of 2. Viral titer and viral copies of infected cells were determined at the indicated time points by measuring TCID50 on DEF cells. All titrations were carried out in three independent experiment. The titers and copies obtained were averaged, and the standard error of the mean was calculated each time point. (**A**) Viral copies. (**B**) Viral titer. (**C**) Viral copies and titer. (**D**) Genome/TCID_50_ ratios.

DEFs were infected with corresponding viruses at an MOI of 2; moreover, DEFs transfected with pcDNA3.1(+)-FLAG-US10 were infected with ΔUS10 to investigate whether exogenous US10 recovered the replication defect of the mutant. Viral genome copies among the four groups showed no significant differences during a 24 h period (Fig. 4A,C), indicating that US10 deletion caused no defect in viral genome replication. For viral titer, no significant differences were observed within 12 h because no mature viruses were produced. The viral titer showed a rising trend at 18 h.p.i, and there was a significant difference between BAC-G and ΔUS10; this difference became more significant at 24 h.p.i. The viral titer of ΔUS10 cultured in DEFs expressing exogenous US10 showed a slight recovery, but it was still significantly lower than that of BAC-G at 24 h.p.i (Fig. 4B,C). As with multistep replication analyses, the genome/TCID_50_ ratios of ΔUS10 were still higher than those of the other groups (Fig. 4D). These results showed that deletion of DEV US10 had no effect on genome replication but strongly impaired infectious virion production.

In general, DEV US10 is non-essential for viral replication, but it plays an important role in viral maturation.

### Regulation of immune-related gene transcription in DEFs by DEV US10

The CCHC-type zinc finger domain in DEV US10 prompted us to perform further analyses of this gene^22,53,54^. One hypothesis is that US10 plays a role in the immune modulation of host cells^59,60^. DEFs were infected with BAC-G and ΔUS10 at an MOI of 2 or mock infected. Total RNA was collected and extracted at 6, 12 and 18 h.p.i. Reverse-transcription cDNAs were used as templates for relative real-time quantitative PCR.

**Figure 5:**
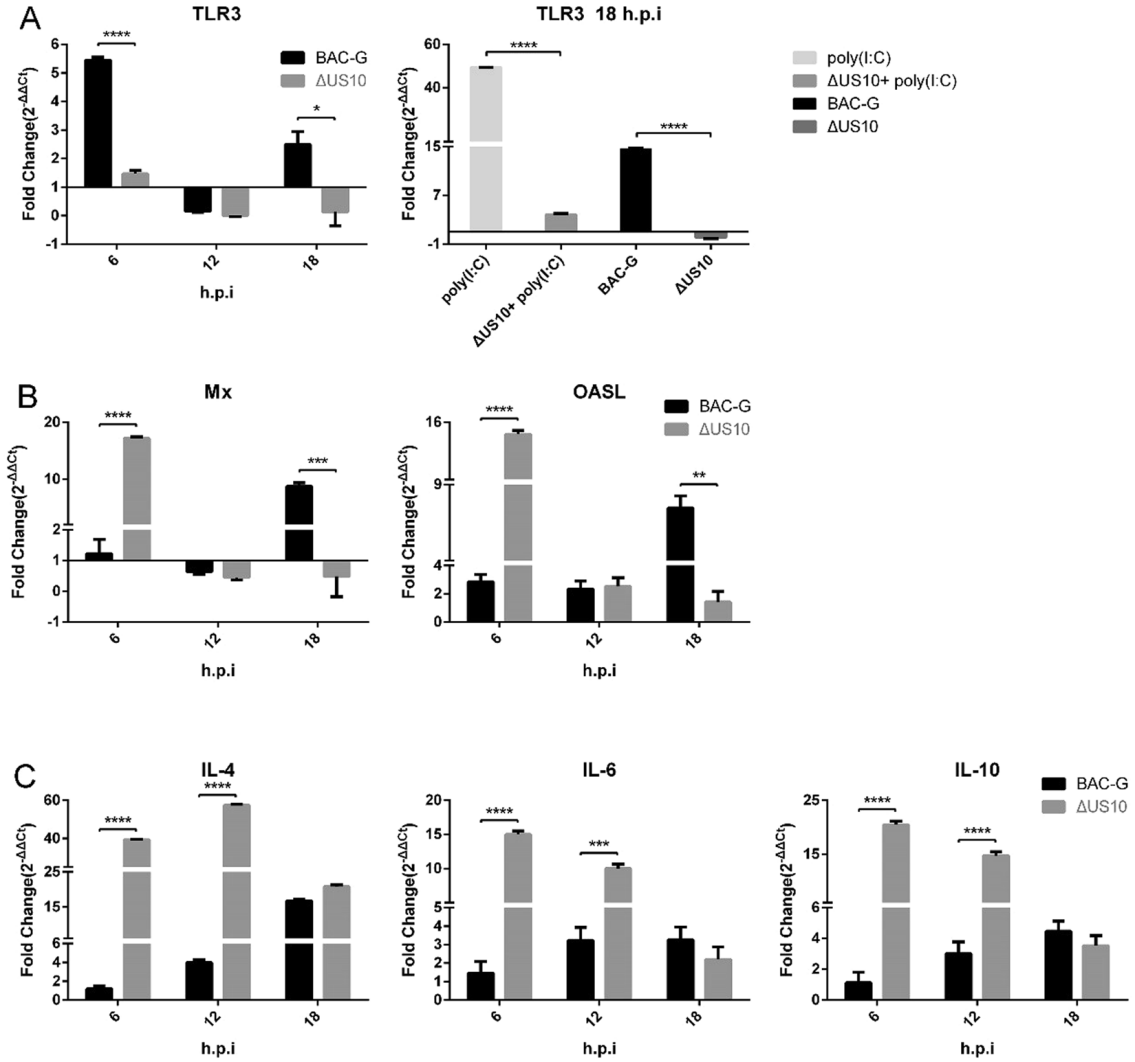
Transcription level of immune-related genes in virus-infected DEFs. Total RNA was collected and extracted at the indicated time points for reverse transcription, and cDNAs were used for qPCR detection. The relative expression levels of immune-related genes were calculated by the 2^−ΔΔCt^ method. Statistical significance was analysed using Student’s *t* test and considered significant as follows: *P < 0.05, **P < 0.01, ***P < 0.0005, ****P < 0.0001.

Transcription levels of some immune-related genes (TLR3, Mx, OASL, IL-4, IL-6 and IL-10 were shown in this paper) and one housekeeping gene (β-actin) were determined by qPCR. TLR3 was significantly upregulated by BAC-G at 6 h.p.i, while TLR3 in ΔUS10-infected DEFs remained at a normal level. At 18 h.p.i, TLR3 was downregulated by ΔUS10, but BAC-G still upregulated TLR3 (Fig. 5A). A verification experiment was then carried out to further test whether ΔUS10 could downregulate TLR3. DEFs were transfected with 1 μg poly(I:C) to activate TLR3 expression, and then, treated cells were mock infected or infected with ΔUS10. DEFs were also infected with BAC-G or ΔUS10 as a control. As a result, poly(I:C) stimulation caused an approximately 49-fold upregulation of TLR3, while ΔUS10-infected treated cells showed only a 4-fold upregulation. Meanwhile, similar results were obtained in virus-infected groups; BAC-G upregulated TLR3, while ΔUS10 downregulated TLR3 (Fig. 5A). Remarkably, the antiviral genes Mx and OASL were significantly upregulated by BAC-G and ΔUS10 at different time points (Fig. 5B). For interleukins, the transcription level of IL-2 showed no difference after US10-deletion (data not shown). IL-4, IL-6 and IL-10 expression remained normal at 6 h.p.i and gradually increased during BAC-G replication, but the transcription levels of IL-4, IL-6 and IL-10 in ΔUS10-infected DEFs were upregulated at all time points (Fig. 5C). These results showed that deletion of US10 caused different transcription trends of some immune-related genes.

## Discussion

Tegument proteins of alphaherpesviruses perform or mediate diverse functions during the viral life cycle, such as nucleocapsid transportation^1,9^, regulation of the host cell immune system^12^, tegumentation and secondary envelopment^1,13^. With the development of reverse genetics techniques, gene deletion has become the most convincing way to study viral gene functions. In a previous study, HSV-1 US10 was characterized as a capsid/tegument-associated phosphoprotein that copurifies with the nuclear matrix, and no further study on the role of US10 in viral replication was reported. As a homologue of HSV-1 US10, DEV US10 is a true late (γ2) gene and encodes a poorly understood tegument protein^22^. To gain insight into the function of DEV US10, we generated US10 deletion and revertant mutants based on the infectious BAC clone of the DEV CHv strain previously constructed by our laboratory^49,58^. Parental and recombinant viruses contain an EGFP marker, which is available for virus rescue observation and precise viral titer determination^55,57,58^.

Here, we report that US10 is non-essential but plays a vital role in viral replication, as the rescued ΔUS10 mutant showed an approximately 100-fold titer reduction at 48 h.p.i in multistep growth kinetic analyses (Figs 2C and 3C). When DEFs were infected with viruses at a low MOI (e.g., 0.02, in this paper), only a few cells were infected at the beginning, and viruses replicated for several rounds until all of the cells were killed. During several rounds of replication, the defect caused by gene deletion was prominently displayed. Then, we observed that the genome copies of ΔUS10 showed only a 4-fold reduction, which was much lower than that of the viral titer. Herpesvirus genome replication is known to occur in the nucleus, and a previous study showed that DEV US10 was located in the cytoplasm^22^. Considering these two points, we speculated that the deletion of US10 caused no defect in viral genome replication. However, the genome copies of ΔUS10 showed a reduction (Fig. 3C). One logical explanation for this apparent discrepancy is that the defect of infectious virion production limits the number of replicating genomes in the next replication cycle.

To further investigate the role of US10 in viral genome replication and infectious virion production, we performed one-step growth kinetic analyses. DEFs were infected with viruses at a high MOI (e.g., MOI of 2, in this paper); theoretically, all the cells became infected at once. The viral titer of all groups showed no change at 6 and 12 h.p.i because herpesviruses require approximately 18 h to complete a life cycle^61^ (Fig. 4B,C). The viral titer increased remarkably at 18 h.p.i., while the immature viruses became infectious. The ΔUS10 mutant still showed a 5- to 20-fold reduction in viral titer after one complete life cycle, while the genome copies of all groups showed no significant differences (Fig. 4A–C). This speculation was proven to be correct, and the replication defect of the ΔUS10 mutant did not affect genome replication. It is possible that US10 either directly or indirectly impairs viral maturation. To examine this possibility, we performed TEM to observe the virion structure of replicating BAC-G and ΔUS10. No immature virions were observed in virus-infected cells (data not shown), indicating that the defect in some aspect of ΔUS10 assembly was hard to visualize via electron microscopy. The deletion of US10 caused an up to 100-fold defect in viral titer, but ΔUS10 still maintained the ability to infect DEFs and showed no observed defect in viral assembly. Our findings indicate that US10 plays a key role in DEV titer but is not indispensable for viral infection.

Zinc finger proteins generally function in DNA- and RNA-binding and regulate DNA transcription or RNA metabolism^59,62,63^. Recent studies revealed the important roles of zinc finger proteins in immune responses^59^, but the function of these proteins in the virus is not well understood. A CCHC-type zinc finger domain was found in DEV US10, and thus, it is tempting to speculate that US10 may function in the interplay between the virus and the cell immune system. TLR3 recognizes double-stranded RNA (dsRNA) and activates innate immunity against pathogen infection. As an erroneous product, dsRNA is produced by converging bidirectional transcription when DEV replicates^64^. TLR3 was upregulated by BAC-G and downregulated by ΔUS10 (Fig. 5A), indicating that US10 might be associated with dsRNA recognition. The transcription trends of antiviral genes (Mx and OASL) and interleukins (IL-4, IL-6 and IL-10) were quite different between BAC-G and ΔUS10-infected DEFs. All the five genes showed transcriptional upregulation at the early stage (6 h.p.i) of ΔUS10 infection. Surprisingly, the transcription levels of immune-related genes in BAC-G and ΔUS10-infected cells were substantially different, there were also some other immune-related genes detected showed no transcription difference (e.g. IL-2), and the underlying mechanism is unclear and needs to be further investigated. In summary, a remarkable defect in DEV replication was shown in the absence of US10, and several immune-related genes in virus-infected cells showed different dynamic transcription levels after US10 deletion. We believe that the data in this paper will supply fundamental information for functional analyses of US10 and DEV pathogenesis.

## Materials and Methods

### Cells and viruses

Monolayer DEFs derived from 9-day-old Cherry Valley duck embryos were cultured in modified Eagle’s medium (MEM) supplemented with 10% newborn bovine serum (NBS). Parental DEV (DEV CHv-BAC-G) with an enhanced green fluorescent protein (EGFP) expression cassette and its BAC clone were generated in our laboratory previously^49,58^.

### Plasmids and antibodies

Rabbit polyclonal antibodies against US10 were prepared in our laboratory, and the pcDNA3.1(+)-FLAG-US10 expression plasmid was constructed as described previously^22^. Mouse polyclonal antibodies against β-actin were purchased from Bioss (China).

### Generation of recombinant viruses

The DEV US10 deletion and revertant mutants were generated by two-step RED recombination^65,66^ using *E. coli* DH10B containing pBAC-DEV, an infectious DEV BAC clone, as described previously^58^. Briefly, the kanamycin-resistant (kanR) gene expression cassette in pKD4 was PCR amplified and electroporated into DH10B cells containing the pBAC-DEV clone and pKD46. RED recombination was mediated by the expression of L-arabinose-induced genes in pKD46, kanR was flanked by two FRT sites, and homologous sequences were introduced into the target sequence, replacing the US10 ORF. Then, the kanR-containing clone was cultured at 42 °C to lose pKD46, and pCP20 was electroporated to induce recombination between two FRT sites. The positive clone was identified by PCR and confirmed by sequencing. For the revertant mutant construction, an almost identical procedure was carried out, except for the use of the kanR-amplification primer 5′ACAAGCGCCAGGATCCGAATAAAGTTCCTCTGTCAGACTACGATGACTCTGACTGAGTGTAGGCTGGAGCTGCTTC-3′. The recombinant BAC plasmids were extracted using the Qiagen Plasmid Midi Kit and transfected into DEFs. Cells were cultured for 5–7 days and harvested until a large amount of green fluorescence with matching CPE was observed. The obtained viruses were named DEV CHv-BAC-G-ΔUS10 (ΔUS10) and DEV CHv-BAC-G-US10FRT (US10FRT), both of which were confirmed by PCR and western blotting, ensuring the deletion and expression of US10.

### Viral replication kinetic determination

For multistep viral replication kinetic analyses, DEFs in 24-well plates were infected with BAC-G, ΔUS10 or US10FRT at an MOI of 0.02. Samples of supernatant and cytoplasm were collected separately at 12, 24, 48, 72, 96 h.p.i and stored at −80 °C, before which cytoplasm samples underwent 3 freeze-thaw cycles. The viral titer determination was performed in three independent repeat TCID_50_ assays.

For one-step viral replication kinetic analysis, an almost identical procedure was carried out, with the following changes: DEFs were infected with the corresponding viruses at an MOI of 2, and samples were collected at 6, 12, 18, and 24 h.p.i.

Real-time quantitative PCR was utilized to derive the number of viral genomes within replication kinetic samples. The primers and probe for qPCR were designed previously in our laboratory to detect DEV UL30. A total of 100 μl of each sample was used for viral DNA extraction, and 1 μl of purified DNA was used for TaqMan PCR analysis.

### Transcription level of immune-related genes in virus-infected DEFs

DEFs in 12-well plates were infected with BAC-G or ΔUS10 at an MOI of 2 or mock infected. Total RNA was collected and extracted at 6, 12, and 18 h.p.i. Reverse transcription was performed according to the instructions of the PrimeScript™ RT reagent Kit with gDNA Eraser (Perfect Real Time, TaKaRa). The cDNAs were used as templates for real-time quantitative PCR. The relative transcription levels of immune-related genes were calculated using the 2^−ΔΔCt^ method^67^. In addition, activation of TLR3 in DEFs was accomplished by poly(I:C)-transfection using Lipofectamine™ 3000 Transfection Reagent.

## Electronic supplementary material

Supplementary Information
