# Supplementary material for: US10 Protein Is Crucial but not Indispensable for Duck Enteritis Virus Infection in Vitro
Source: Sci Rep. 2018 Nov 7;8:16510. doi: 10.1038/s41598-018-34503-7 (PMC6220328; doi:10.1038/s41598-018-34503-7)
Supplement: Supplementary file 1 — Supplementary Information [file 41598_2018_34503_MOESM1_ESM.pdf]

US10 Protein Is Crucial but not Indispensable for Duck Plague Virus Infection in Vitro

Yunchao Ma [1,2¶], Qiurui Zeng[4¶],Mingshu Wang[1,2,3¶], Anchun Cheng[1,2,3*], Renyong Jia[1,2,3], Qiao Yang[1,2,3], Ying Wu[1,2,3], Xin-Xin Zhao[1,2,3], Mafeng Liu[1,2,3], Dekang Zhu[2,3], Shun Chen[1,2,3], Shaqiu Zhang[1,2,3],Yunya Liu[1,2,3], Yanling Yu[1,2,3], Ling Zhang[1,2,3], Xiaoyue Chen[1,2,3]

[1]Institute of Preventive Veterinary Medicine, Sichuan Agricultural University, Wenjiang, Chengdu City, Sichuan, 611130, P.R. China
[2]Key Laboratory of Animal Disease and Human Health of Sichuan Province, Sichuan Agricultural University, Wenjiang, Chengdu City, Sichuan, 611130, P.R. China
[3]Avian Disease Research Center, College of Veterinary Medicine, Sichuan Agricultural University, Wenjiang, Chengdu City, Sichuan, 611130, P.R. China
[4]School of medicine, Shanghai Jiao Tong University, 200025, P.R. China

[¶]These authors contributed equally to this work as first authors.

Corresponding authors: Anchun Cheng(chenganchun@vip.163.com)

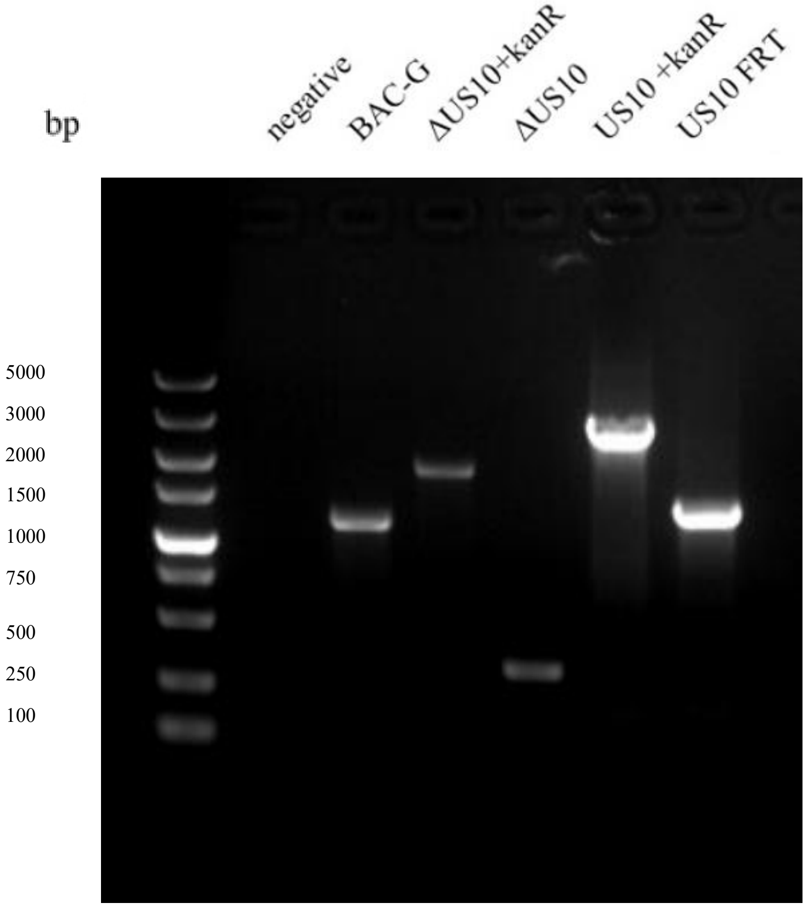
bp
negative
BAC-G
ΔUS10+kanR
ΔUS10
US10 +kanR
US10 FRT
5000
3000
2000
1500
1000
750
500
250
100

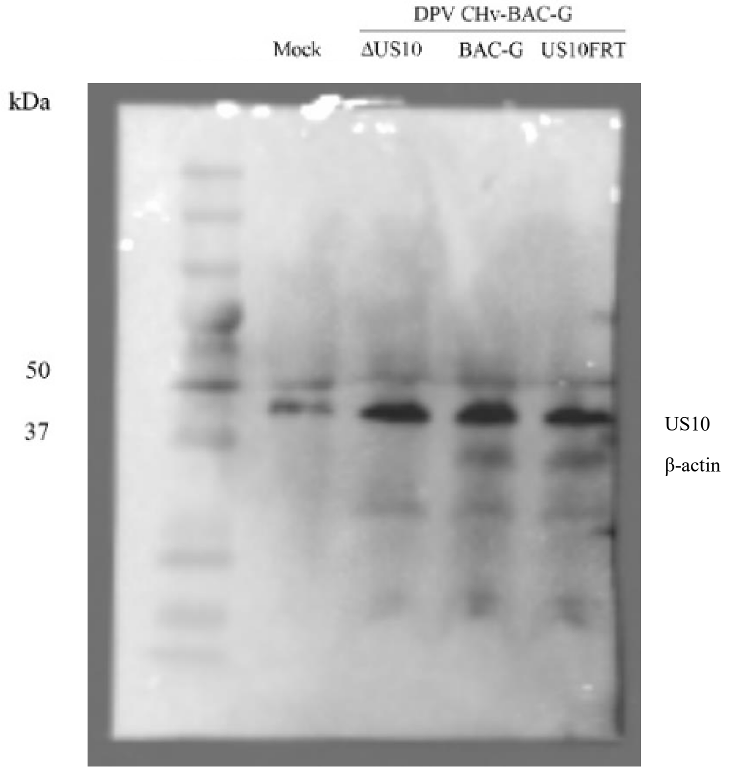
DPV CHv-BAC-G
Mock
ΔUS10
BAC-G
US10FRT
kDa
50
37
US10
β-actin
